# mTORC1 Overactivation as a Key Aging Factor in the Progression to Type 2 Diabetes Mellitus

**DOI:** 10.3389/fendo.2018.00621

**Published:** 2018-10-16

**Authors:** Carlos Guillén, Manuel Benito

**Affiliations:** ^1^Department of Biochemistry and Molecular Biology, Faculty of Pharmacy, Complutense University of Madrid, Madrid, Spain; ^2^Spanish Biomedical Research Centre in Diabetes and Associated Metabolic Disorders (CIBERDEM), Instituto de Salud Carlos III, Madrid, Spain

**Keywords:** T2DM, autophagy, amylin, mitophagy, mTORC1, TSC2

## Abstract

Type 2 Diabetes Mellitus (T2DM), a worldwide epidemics, is a progressive disease initially developing an insulin resistant state, with manifest pancreatic beta islet overwork and hyperinsulinemia. As the disease progresses, pancreatic β cells are overwhelmed and fails in their capacity to compensate insulin resistance. In addition, it is usually associated with other metabolic diseases such as hyperlipidemia, obesity and the metabolic syndrome. During the progression to T2DM there is a chronic activation of mTORC1 signaling pathway, which induces aging and acts as an endogenous inhibitor of autophagy. The complex 1 of mTOR (mTORC1) controls cell proliferation, cell growth as well as metabolism in a variety of cell types through a complex signaling network. Autophagy is involved in the recycling of cellular components for energy generation under nutrient deprivation, and serves as a complementary degradation system to the ubiquitin-proteasome pathway. Autophagy represents a protective mechanism for different cell types, including pancreatic β cells, and potentiates β cell survival across the progression to T2DM. Here, we focus our attention on the chronic overactivation of mTORC1 signaling pathway in β islets from prediabetics patients, making these cells more prone to trigger apoptosis upon several cellular stressors and allowing the progression from prediabetes to type 2 diabetes status.

## mTORC1 signaling network: upstream and downstream effectors

Tuberous sclerosis complex (TSC) is an essential regulator of mTORC1 signaling pathway. TSC is a heterotrimer protein complex formed by the association of TSC1 (hamartin), TSC2 (tuberin) and Tre2-Bub2-Cdc16-1 domain family member 7 (TDC1D7) (1). This complex is the major inhibitory node of the mechanistic target of rapamycin complex 1 (mTORC1) (2). TSC1-TSC2-TBC1D7 complex exerts its inhibitory effect on mTORC1 by a member of the Ras superfamily, called Rheb. TSC2 presents a GTPase activating domain (GAP) favoring the transformation of GTP-Rheb (ras homology enriched in brain) into GDP-Rheb, avoiding mTORC1 activation (2–5). TSC1 and TSC2 are modulated by multiple mechanisms, including phosphorylation. TSC1/TSC2 integrates signals coming from the energetic status of the cell and nutritional availability, with those from extracellular signaling coming from hormones or growth factors such as insulin or IGF1 respectively (2, 6).

mTOR (mechanistic target of rapamycin) is a serine-threonine kinase, belonging to the phosphatidyl-inositol-3 kinase related kinases (PIKK) (7). Depending on the proteins that are associated with mTOR, it can be found into two different complexes, mTORC1 and mTORC2. mTORC1 is the “rapamycin-sensitive” complex and mTORC2 is the “rapamycin-independent” complex. There are two specific components of mTORC1, the regulatory-associated protein of mTOR (RAPTOR) and the 40-Kda proline-rich Akt substrate (PRAS40) (8–10). However, the rapamycin-independent companion of mTOR, known as RICTOR, the mammalian stress-activated MAP kinase-interacting protein 1 (mSIN1) and the protein observed with RICTOR (PROTOR) are specific members of mTORC2 complex (11). The best characterized substrates of mTORC1 are S6 kinase 1 (S6K1) and eIF4E-binding protein 1 (4E-BP1), controlling protein synthesis and ribosome biogenesis (12). Activation of mTORC2 leads to phosphorylation and activation Akt at serine 473. mTORC2 it is considered as the PDK2 required for the full activation of Akt (13–15).

In the last years, it has been determined that mTORC1 activation is produced on the lysosomal membrane through a complex mechanism involving different proteins (16). Phosphorylation events coming from growth factors, through the Akt and MAPK kinases, directly phosphorylates TSC2 and dissociates it from the surface of the lysosome, avoiding GAP activity toward Rheb and mTORC1 activation (17). Furthermore, nutrients such as aminoacids are capable to activate mTORC1 signaling in a Rag-dependent manner, another group of small G-proteins, acting as heterodimers for mTORC1 activation (18, 19). The presence of aminoacids affects RagA/B-GTP ratio vs. RagA/B-GDP through GAP activity of GATOR1 (20) and the guanine exchange factor activity (GEF) of the complex called “Ragulator” (21). Then, mTORC1 is regulated by two different systems of small G proteins, named Rag and Rheb, on the surface of the lysosome (16). Our group has demonstrated that TSC2, apart from its phosphorylation state, can be regulated by acetylation in different lysine residues (22). Now, we are analyzing the relevance of acetylation status of TSC2 for its recruitment to the membrane of the lysosome.

Another important effector of mTORC1 signaling is AMP-activated protein kinase (AMPK). AMPK is highly conserved kinase in eukaryotes. AMPK acts as an allosteric sensor of cellular energy status and regulates anabolic and catabolic signaling pathways for the maintenance of energy homeostasis (23, 24). In addition, AMPK inhibits mTORC1 signaling directly by TSC2 phosphorylation, favoring TSC1-TSC2 association (25). Furthermore, AMPK modulates mTORC1, independently from TSC2 by raptor phosphorylation and inactivation of mTORC1 (26). Moreover, AMPK and autophagy are connected directly by phosphorylation of ULK1 that leads to autophagy induction. In fact, several residues of ULK1 protein are directly phosphorylated by AMPK (27, 28). It has recently been proposed that ULK1 acetylation is essential for autophagy activation (29, 30).

## Autophagy and its relevance in pancreatic β cells

Autophagy is a process that controls cytoplasm quality by the elimination of protein aggregates or altered organelles (31, 32). Autophagy is involved in the shutdown of energy-consuming pathways and the stimulation of catabolic processes through the degradation of cellular components under nutrient deprivation (33). Alternatively, to the ubiquitin-proteasome system (UPS), cells depend on autophagy as an alternative degradation system. Furthermore, autophagy it is involved in the elimination of misfolded proteins or altered organelles. The term autophagy encompasses several conserved mechanisms in eukaryotes that can be classified as: macroautophagy, chaperone-mediated autophagy and microautophagy. Chaperone-mediated autophagy consists in the recognition of a specific sequence by a chaperone (hsc70) and then migration of the complex to the lysosome. There, the complex interacts with a receptor in the lysosomal membrane, called LAMP-2A, translocates inside and it is finally degraded (34). In microautophagy, the lysosome directly engulfs the components that are going to be degraded (35). As a general process, macroautophagy it is characterized by the production of a double membrane compartment, termed autophagosome, which surrounds cytoplasmic components and then, fuses with lysosomes where proteolytic activity of the later will degrade the engulfed components (36). Autophagy protects pancreatic β cells, increasing pancreatic β cell survival in the progression to T2DM (37, 38). Using a mouse model Atg-7 deletion specifically in pancreatic β cells, it has been determined the essential implication of autophagy in the survival of pancreatic β cells (37). Furthermore, our group has defined this protection of autophagy in ER-stress using a model of insulin secretion-deficient β cells (39). The protective role of autophagy has been determined for avoiding the toxicity of human amylin (hIAPP) (40, 41). In fact, a mouse model with a chronic overactivation of mTORC1 (β-TSC2-/-) showed an increase in pancreatic β cell death and an impairment in autophagy, as we demonstrated in collaboration with Yoshiaki Kido's lab in Kobe University (42). It has recently been observed in human pancreatic β cells an association between dysregulation in autophagy with an increased in cell death (43, 44). Accordingly, this data point to mTORC1 as a signaling pathway that is overactivated in diabetic patients and, at least partially responsible of pancreatic beta cell failure. All the data exposed in the present paper are derived from animal models and not from pre-diabetic patients. There is a clear effect of mTORC1 hyperactivation and the progression to T2DM, but in animal models. The extrapolation to the possible effect in humans has to be assured.

## mTORC1 and mitophagy. consequences on T2DM

**Figure 1:**
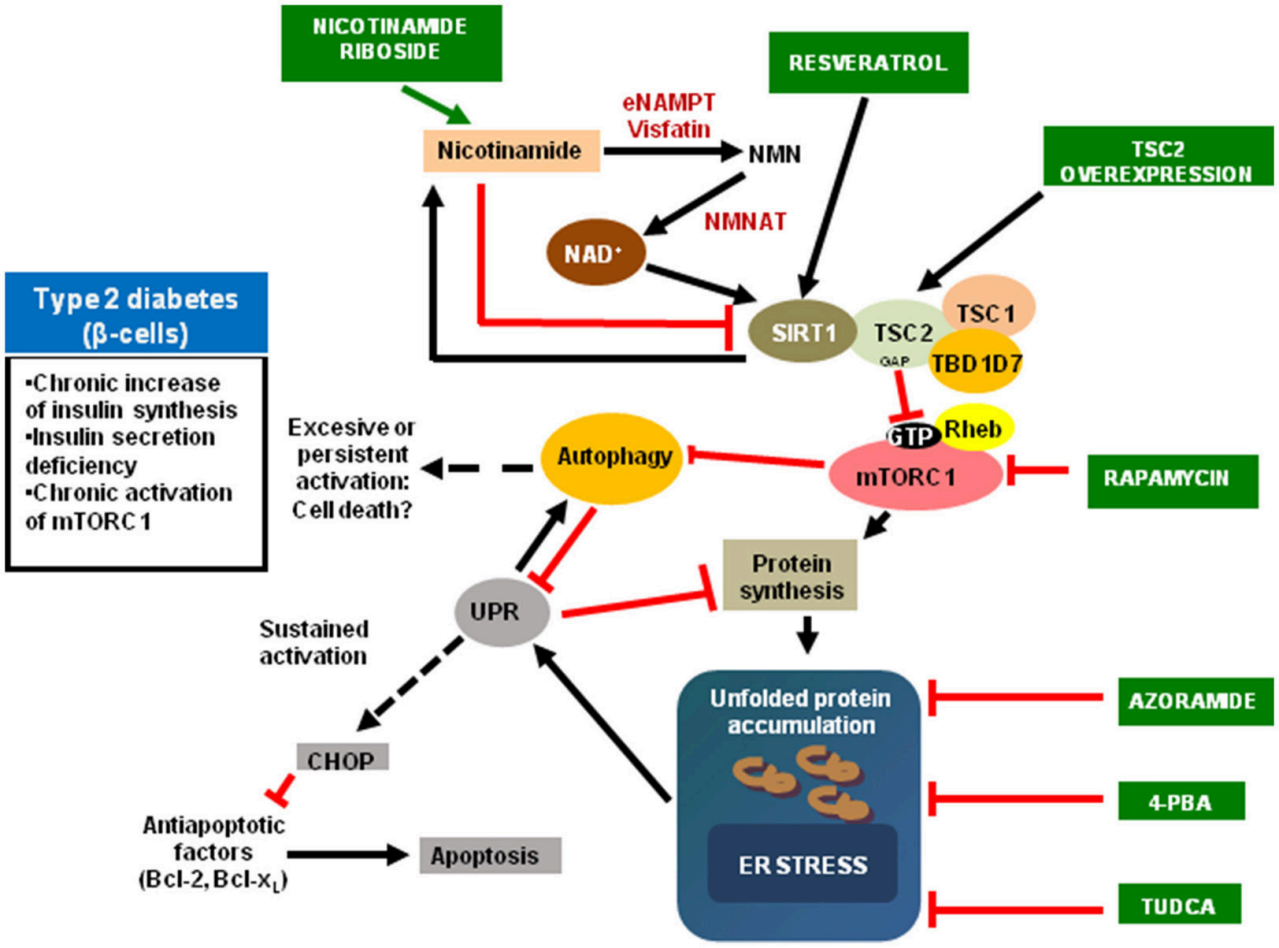
Scheme depicting the main upstream and downstream regulators of mTORC1 signaling pathway. The possible agents that affect aging process are in green boxes. eNAMPT, extracellular nicotinamide phosphoribosyltransferase; NMNAT, Nicotinamide mononucleotide adenylyltransferase; 4-PBA, 4-phenylbutyric acid; TUDCA, Tauroursodeoxycholic acid; CHOP, CCAAT-enhancer-binding protein homologous protein.

mTORC1 it is an endogenous inhibitor of autophagy. Apart from the bulk autophagy, a non-specific mechanism, all the organelles are submitted to a specific degradation. In the case of mitochondria, this process is named mitophagy. Additionally, a mitochondrial unfolded protein response (mtUPR) for the degradation of protein aggregates in different locations inside the mitochondria, such as the matrix and in the inner membrane, occurs (45, 46). The inhibition of mTORC1 slows aging by an increased in autophagy, favoring the elimination of misfolded proteins and impaired organelles such as mitochondria, avoiding its accumulation, and associated with aging and different aging-related diseases such as T2DM, or Parkinson disease, or Alzheimer disease (47). In fact, in the progression to T2DM exists an increasing alteration in mitochondrial morphology and function. Then, an increased in mitophagy could be a potential mechanism for delaying the progression to the disease and maintain a preserved β-cell function (48). Mitophagy is a complex process and there are several possible mechanisms. During the development and maturation of red blood cells, it is necessary a removal of mitochondria in the precursor cell or reticulocyte through a NIP3-like protein X (NIX, also known as BNIP3L) mechanism (49). However, in mammals exist another mechanism with involves the system PTEN-induced putative kinase 1 (PINK1)-Parkin pathway. PINK1 is a serine-threonine kinase recruited to the healthy mitochondria, which is imported into the TIM complex of the inner membrane, where it is cleaved by the mitochondrial processing peptidase (MPP) (50) and presenilin-associated rhomboid-like protein (PARL) (51, 52). PINK1 accumulates specifically in damaged mitochondria, because the processing system is inhibited. Then, the E3-ubiquitin ligase Parkin it is recruited to the outer membrane (OM) of the mitochondria, ubiquitinates different substrates, driving the mitophagic process (53). Our group has determined that in MEF TSC2 KO, which presents a hyperactivation of mTORC1, there is an impairment in the PINK1 recruitment in response to a mitophagic inducer such as carbonyl cyanide m-chlorophenylhydrazone (CCCP). Furthermore, in TSC2–/– cells there is a reduction in *Pink1* expression as well as PINK1 protein production. Then, in cells lacking TSC2, an accumulation of damaged and aged mitochondria occurs (54). This situation contributes to the accumulation of reactive species of oxygen (ROS) as occurred in diabetics (48). In addition, amylin accumulates and forms cytotoxic oligomers, which damage the cell membrane and directly affects to the functionality and viability of β cells (55). We have recently published that INS1E overexpressing human amylin (INS1E-hIAPP) presents a hyperactivation of mTORC1 and a diminished level of TSC2. Furthermore, these cells possess an impairment in mitophagic flux with the concomitant accumulation of damaged mitochondria (56). However, the mechanism underlying pancreatic β cell toxicity during the formation of amyloid remains unknown (57). In Figure 1, it is depicted the main players in the regulation of pancreatic β cell fate and different drugs acting at different levels.

## mTORC1, aging and its effects on T2DM

It is well known that insulin signaling is involved in the control of longevity in a wide spectrum of organisms including worms, flies, and mice (58–62). In addition, the use of rapamycin or knocking-down mTOR or S6K1 can promote life extension in several species (63–66). In addition, TSC1 and TSC2 activation, which negatively controls mTORC1, prolongs life span in *Drosophila* (67). During aging or under a hypercaloric diet exists an mTORC1 hyperactivity, which derives into a disruption in autophagy and, concomitantly an increase in ER stress (16). In this regard, nicotinamide adenine dinucleotide (NAD^+^) as well as sirtuins levels can be modulated during caloric restriction, activating autophagy, and directly affecting mammalian longevity (68). Mammalian sirtuins are composed of seven members (SIRT1-7) and can be activated by several stimuli including energy deprivation, caloric restriction and resveratrol (69, 70). Sirtuins are a group of NAD^+^-dependent histone deacetylases with homology to the yeast silent information regulator 2 (Sir2) proteins (71). SIRT1-3 catalyzed deacetylation reaction producing a by-product, nicotinamide and O-acetyl ADP-ribose (OAADPr), along with the deacetylated lysine as it has been reviewed in (72). NAD^+^ levels declines in different tissues with aging, altering the functionality of sirtuins and hence plays a key role in multiple diseases, such as metabolic disorders, neurodegenerative diseases and many others (73). Then, NAD^+^ plays a central role in aging process and longevity (74).

Sirtuins can be activated by different treatments, such as caloric restriction, rapamycin, resveratrol and many others. In fact, all of these treatments are capable to modulate mTORC1 signaling (75). The overactivation of mTORC1 signaling specifically in pancreatic β cells leads to an augmented in β cell mass, which are related to hyperinsulinemia and hypoglycemia (76–79). However, chronic overactivation of mTORC1 signaling pathway develops a progressive hyperglycemia and a diminished islet mass (76). In this regard, we recently published that overactivation of mTORC1 in beta islets from a β-cell specific deletion of Tsc2 (βTsc2 (–/–) mice) induces an impairment in autophagy with an accumulation of damaged mitochondria. Those deleterious effects were partially reverted by the use of rapamycin (42).

In pancreatic β cells, SIRT1 represses the expression of uncoupling protein 2 (UCP2), leading to an increase in mitochondrial ATP production, thus enhancing insulin secretion (80). Accordingly, a β cell-specific SIRT1-overexpressing transgenic mice (BESTO) has a better glucose tolerance and a boost in insulin secretion (81). Nowadays, SIRT1 it is considered a regulator of different metabolic pathways. There are multiple SIRT1-regulated targets, modulating transcriptional activity of different transcription factors, such as peroxisome proliferator-activated receptor gamma (PPARγ), PPARα, PPAR gamma coactivator 1 alpha (PGC-1α), and the forkhead box, subgroup O (FOXO) family. Then, SIRT1 is capable to regulate different processes like insulin secretion, gluconeogenesis and fatty acid oxidation (82).

## Type 2 diabetes mellitus and mTORC1

Type 2 diabetes mellitus (T2DM) is a very complicated disorder and it is considered epidemic in the world (83). T2DM it is a progressive disease including insulin resistance, β-cell hyperplasia and/or β cell hypertrophy, that mediates a compensatory insulin secretion and subsequently hyperinsulinemia, pancreatic β cell dysfunction and a loss of cell identity or de-differentiation (84). However, the underlying mechanism mediating pancreatic β cell fate in type 2 diabetics is not completely known. T2DM is associated with higher production of glucose and lipids facilitating β cell death. Furthermore, advanced glycation end-products (AGEs) have been proposed as pancreatic β cell death inducers (85). The hyperamylinemia found in obese people and in insulin-resistant patients, contributes to its oligomerization inside pancreatic β cells, being deleterious for pancreatic β cells (86). From the whole amount of autopsy from patients with T2DM, around 80% present amyloid deposits in the pancreas. However, the importance of amylin it is not yet completely understood. Thus, at the insulin resistant prediabetic stage, mTORC1 is a key effector for the growth and survival of pancreatic β cells. However, if mTORC1 remains chronically overactivated, pancreatic beta cell death occurs and the compensatory insulin secretion mechanism it is compromised. Then, mTORC1 is a double-edged sword in the progression to T2DM (87). The location where protein synthesis occurs is in the endoplasmic reticulum (ER). When misfolded proteins are accumulated into the ER, the unfolded protein response (UPR) is activated. UPR is a protective mechanism that alleviates the cell from that overload. However, if UPR activation it is maintained for a long period of time, pancreatic β cell death occurs (88). T2DM alters the capacity of ER to manage the increased demand of protein synthesis of pancreatic β cells (89). Increasing the expression of endogenous chaperones such as Bip (90), the use of chemical chaperones, such as taurine-conjugated ursodeoxycholic acid (TUDCA) or 4-Phenyl butyric acid (4-PBA) (91), or increasing the ER protein folding capacity by the use of azoramide (92), diminish β cell failure and facilitate the proper folding and avoiding protein aggregation and accumulation of damaged organelles.

However, mTORC1 hyperactivation is observed in other tissues, such as in the heart muscle contributing to the dysfunction and complication of T2DM (93). Furthermore, not only in cardiac tissue, in the liver, apart from being involved in the regulation of lipid homeostasis, facilitating lipogenesis and inhibiting lipolysis and lipophagy (94), is upregulated in insulin resistant states, such as T2DM contributing to the dysregulation of glucose as well as lipid homeostasis (95). In adipose tissues (96), mTORC1 is also involved in the generation of insulin resistance as it has been reviewed in (16).

## Concluding remarks

Diabetes is a multifactorial and progressive disease with two phases; firstly, a prediabetic stage, with an insulin resistance and hyperinsulinemia, and secondly as manifest diabetes associated with hypoinsulinemia and hyperglycemia. Then, it is crucial to understand the transition from prediabetes to type 2-diabetes status and the underlying molecular mechanisms of disease. At this stage, chronic overactivation of mTORC1 signaling pathway in β islets from prediabetics patients leads to on one hand to the expansion of the pancreatic beta cell mass and, on the other to the inhibition of autophagy as protective mechanism of beta cells against the attack of several stressors, making these cells more prone to trigger apoptosis. Thus, the maintenance of a functional autophagy it is an essential component to protect and prolong pancreatic β cell life span precluding chronic hyperglycemia.

## Author contributions

All authors listed have made a substantial, direct and intellectual contribution to the work, and approved it for publication.

### Conflict of interest statement

The authors declare that the research was conducted in the absence of any commercial or financial relationships that could be construed as a potential conflict of interest.
